# Genetic and Clinical Characteristics of Patients in the Middle East With Multisystem Inflammatory Syndrome in Children

**DOI:** 10.1001/jamanetworkopen.2022.14985

**Published:** 2022-05-31

**Authors:** Walid Abuhammour, Lemis Yavuz, Ruchi Jain, Khawla Abu Hammour, Ghalia F. Al-Hammouri, Maha El Naofal, Nour Halabi, Sawsan Yaslam, Sathishkumar Ramaswamy, Alan Taylor, Deena Wafadari, Ali Alsarhan, Hamda Khansaheb, Zulfa Omar Deesi, Rupa Murthy Varghese, Mohammed Uddin, Hanan Al Suwaidi, Suleiman Al-Hammadi, Abdulmajeed Alkhaja, Laila Mohamed AlDabal, Tom Loney, Norbert Nowotny, Abdulla Al Khayat, Alawi Alsheikh-Ali, Ahmad Abou Tayoun

**Affiliations:** 1 Al Jalila Children’s Hospital, Dubai, United Arab Emirates; 2 Al Jalila Genomics Center, Al Jalila Children’s Hospital, Dubai, United Arab Emirates; 3 Department of Biopharmaceutics and Clinical Pharmacy, The University of Jordan, Amman, Jordan; 4 Department of Pediatrics, Specialty Hospital, Amman, Jordan; 5 Medical Education and Research Department, Dubai Health Authority, Dubai, United Arab Emirates; 6 Microbiology and Infection Control Unit, Pathology and Genetics Department, Latifa Hospital for Women and Children, Dubai, United Arab Emirates; 7 Virology Laboratory, Microbiology and Infection Control Unit, Latifa Hospital for Women and Children, Dubai, United Arab Emirates; 8 College of Medicine, Mohammed Bin Rashid University of Medicine and Health Sciences, Dubai, United Arab Emirates; 9 Medical Affairs Department, Rashid Hospital, Dubai Health Authority, Dubai, United Arab Emirates; 10 Institute of Virology, University of Veterinary Medicine Vienna, Vienna, Austria; 11 Dubai Health Authority, Dubai, United Arab Emirates

## Abstract

**Importance:**

Clinical, genetic, and laboratory characteristics of Middle Eastern patients with multisystem inflammatory syndrome in children (MIS-C) have not yet been documented.

**Objective:**

To assess the genetic and clinical characteristics of patients with MIS-C of primarily Arab and Asian origin.

**Design, Setting, and Participants:**

A prospective, multicenter cohort study was conducted from September 1, 2020, to August 31, 2021, in the United Arab Emirates and Jordan. Forty-five patients with MIS-C and a matched control group of 25 healthy children with a confirmed SARS-CoV-2 infection status were recruited. Whole exome sequencing in all 70 participants was performed to identify rare, likely deleterious variants in patients with MIS-C and to correlate genetic findings with the clinical course of illness.

**Exposures:**

SARS-CoV-2.

**Main Outcomes and Measures:**

Fever, organ system complications, laboratory biomarkers, whole exome sequencing findings, treatments, and clinical outcomes were measured. The Mann-Whitney *U* test was used to assess the association between genetic variants and MIS-C attributes. The Fisher exact test was used to compute the genetic burden in MIS-C relative to controls.

**Results:**

A total of 45 patients with MIS-C (23 [51.1%] male; 30 [66.7%] of Middle Eastern origin; mean [SD] age, 6.7 [3.6] years) and 25 controls (17 [68.0%] male; 24 [96.0%] of Middle Eastern origin; mean [SD] age 7.4 [4.0] years) participated in the study. Key inflammatory markers were significantly dysregulated in all patients with MIS-C. Mucocutaneous and gastrointestinal manifestations were each reported in 36 patients (80.0%; 95% CI, 66.1%-89.1%), cardiac findings were reported in 22 (48.9%; 95% CI, 35.0%-63.0%), and neurologic findings were reported in 14 (31.1%; 95% CI, 19.5%-45.6%). Rare, likely deleterious heterozygous variants in immune-related genes, including *TLR3*, *TLR6*, *IL22RA2*, *IFNB1*, and *IFNA6*, were identified in 19 patients (42.2%; 95% CI, 29.0%-56.7%), of whom 7 had multiple variants. There was higher enrichment of genetic variants in patients relative to controls (29 vs 3, *P* < .001). Patients with those variants tended to have earlier disease onset (7 patients [36.8%; 95% CI, 19.1%-58.9%] with genetic findings vs 2 [7.7%; 95% CI, 2.1%-24.1%] without genetic findings were younger than 3 years at onset) and resistance to treatment (8 patients [42.1%; 95% CI, 23.1%-63.7%] with genetic findings vs 3 patients [11.5%; 95% CI, 4.0%-29.0%] without genetic findings received 2 doses of intravenous immunoglobulin).

**Conclusions and Relevance:**

The results of this cohort study suggest that rare, likely deleterious genetic variants may contribute to MIS-C disease. This finding paves the way for additional studies with larger, diverse populations to fully characterize the genetic contribution to this new disease entity.

## Key Points

**Question:**

What are the clinical, genetic, and laboratory characteristics of Middle Eastern patients with multisystem inflammatory syndrome in children (MIS-C)?

**Findings:**

In this cohort study of 45 patients with MIS-C of primarily Arab and Asian origins, an enrichment of rare, likely deleterious immune-related genetic variants was found, with a possible association between genetic findings and MIS-C onset and resistance to treatment.

**Meaning:**

These findings suggest that comprehensive genetic profiling of patients with MIS-C of diverse ethnicities is essential to characterize the genetic contribution to this disease.

## Teaser

This cohort study of patients with multisystem inflammatory syndrome in children assesses whether rare deleterious genetic variants that affect immune-related genes are enriched in patients of Middle Eastern origin.

## Introduction

Multisystem inflammatory syndrome in children (MIS-C) is a critical and potentially life-threatening complication of COVID-19 in pediatric settings. MIS-C was first identified in Europe, initially termed SARS-CoV-2–related pediatric multisystem inflammatory syndrome, and later referred to as MIS-C by the US Centers for Disease Control and Prevention.^1^ Children who were diagnosed with MIS-C tested positive for SARS-CoV-2 infection by reverse transcriptase–polymerase chain reaction (RT-PCR) or antibody testing^2,3,4,5^ and had fever for more than 4 days,^2,6,7,8^ with the most common presenting features including gastrointestinal symptoms (80%-100%), mucocutaneous involvement, neurologic symptoms, conjunctivitis, and cardiac complications.^7,8,9,10^ Epidemiologic, clinical, and immunologic investigations have revealed that MIS-C has phenotypic similarities to Kawasaki disease.^11^ However, immune response in patients with Kawasaki disease is typically characterized by proinflammatory signatures, including increases in interleukin (IL) 17 and relatively less macrophage activation syndrome–like cytokine profile levels, whereas patients with MIS-C have high levels of IL-15 and interferon (IFN) γ in severe cases, and more than 50% of patients with MIS-C have a macrophage activation syndrome–like cytokine phenotype.^12^

Some possible indications of the pathophysiologic mechanisms behind MIS-C have been identified. Cytokine profiling of patients with MIS-C revealed elevated inflammatory markers (IL-18 and IL-6), activation of myeloid and lymphocytic chemokines (CCL3, CCL4, and CDCP1), and dysregulation of mucosal immune markers (IL-17A, CCL20, and CCL28).^13^ Furthermore, B-cell receptor sequencing and autoantibody assays of patients with MIS-C identified dysregulated B-cell responses, autoantibody production, and complement- and myeloid cell–mediated inflammation.^14^ Furthermore, immunologic profiling of children with MIS-C and children with COVID-19 revealed distinct features of lymphocyte activation, mainly CX3CR1^+^ CD8^+^ T-cell activation, in patients with MIS-C.^15^

In a recent study,^16^ exome sequencing of 2 patients with MIS-C delineated a role of 1 gene, *SOCS1* (OMIM 603597), in the IFN pathway in predisposing individuals to infection-associated autoimmune cytopenias. Similarly, another exome sequencing study^17^ identified defects in *XIAP* (OMIM 300079) and *CYBB *(OMIM 300481) in 3 of 18 sequenced patients with MIS-C, whereas RNA sequencing of patients with MIS-C revealed dysregulated cytotoxic lymphocyte responses to SARS-CoV-2 infection as major drivers.^18^ These genetic studies suggest dysregulation of the inflammatory response as a characteristic of MIS-C, although they were limited to a small number of patients who were mostly of European origin.

We performed whole exome sequencing in 45 patients, primarily of Middle Eastern origin, with MIS-C and 25 healthy children who were infected with SARS-CoV-2 but remained asymptomatic or developed only mild clinical symptoms. We hypothesized that rare, deleterious genetic variants affecting immune-related genes would be enriched in patients of Middle Eastern origin with MIS-C compared with matched controls and that such variation would contribute to disease onset, symptoms, clinical outcomes, and/or management.

## Methods

### Study Design and Participants

This cohort study, conducted from September 1, 2020, to August 31, 2021, was approved by the Dubai Scientific Research Ethics Committee—Dubai Health Authority and the institutional review board of the Specialty Hospital, Jordan. Patients (and their guardians) recruited in Dubai or Jordan provided written informed consent for their deidentified data to be used for research, and this study was performed in accordance with the relevant laws and regulations that govern research in both countries. This study followed the Strengthening the Reporting of Observational Studies in Epidemiology (STROBE) reporting guideline.^19^

We recruited 70 children between 1 day and 18 years of age and divided them into 2 groups for this study. The first group included 45 patients diagnosed with MIS-C. Inclusion criteria for this group were consistent with the case definition of MIS-C set by the World Health Organization or Center for Disease Control and Prevention (eTable 1 in the Supplement). All patients had evidence of SARS-CoV-2 infection by RT-PCR, SARS-CoV-2 antibodies, or recent exposure to a confirmed case of COVID-19. At least 2 organs were affected by the illness, and the blood profiles of these patients revealed increased inflammatory markers. Exclusion criteria were applied to all patients with another diagnosis that could affect their disease course, such as congenital heart disease, failure to thrive, or other syndromes. The second group was recruited at the same time and included 25 healthy children (control group) (eFigure 1 in the Supplement) who had a SARS-CoV-2 infection confirmed by RT-PCR but were asymptomatic or experiencing mild symptoms. Individuals in the control group were followed up for 12 weeks to ensure that no signs of MIS-C disease were detected.

### Clinical and Demographic Information

Deidentified patient information was extracted from electronic medical records at Al Jalila Children’s Hospital (Dubai, United Arab Emirates), Latifah Hospital (Dubai, United Arab Emirates), and the Specialty Hospital (Jordan), where those patients were treated and recruited for the study. Variables included demographic information, signs and symptoms on admission, inflammatory markers with cytokine profile, cardiac manifestation, course of illness, admission to the pediatric intensive care unit (PICU), treatment, and outcome. Patient’s country of origin was determined through hospital medical records. This study focused exclusively on patients from Arab countries, which we define as all Middle Eastern (Arab-speaking) countries except Iran and Turkey.

### Whole Exome Sequencing

**Figure 1.**
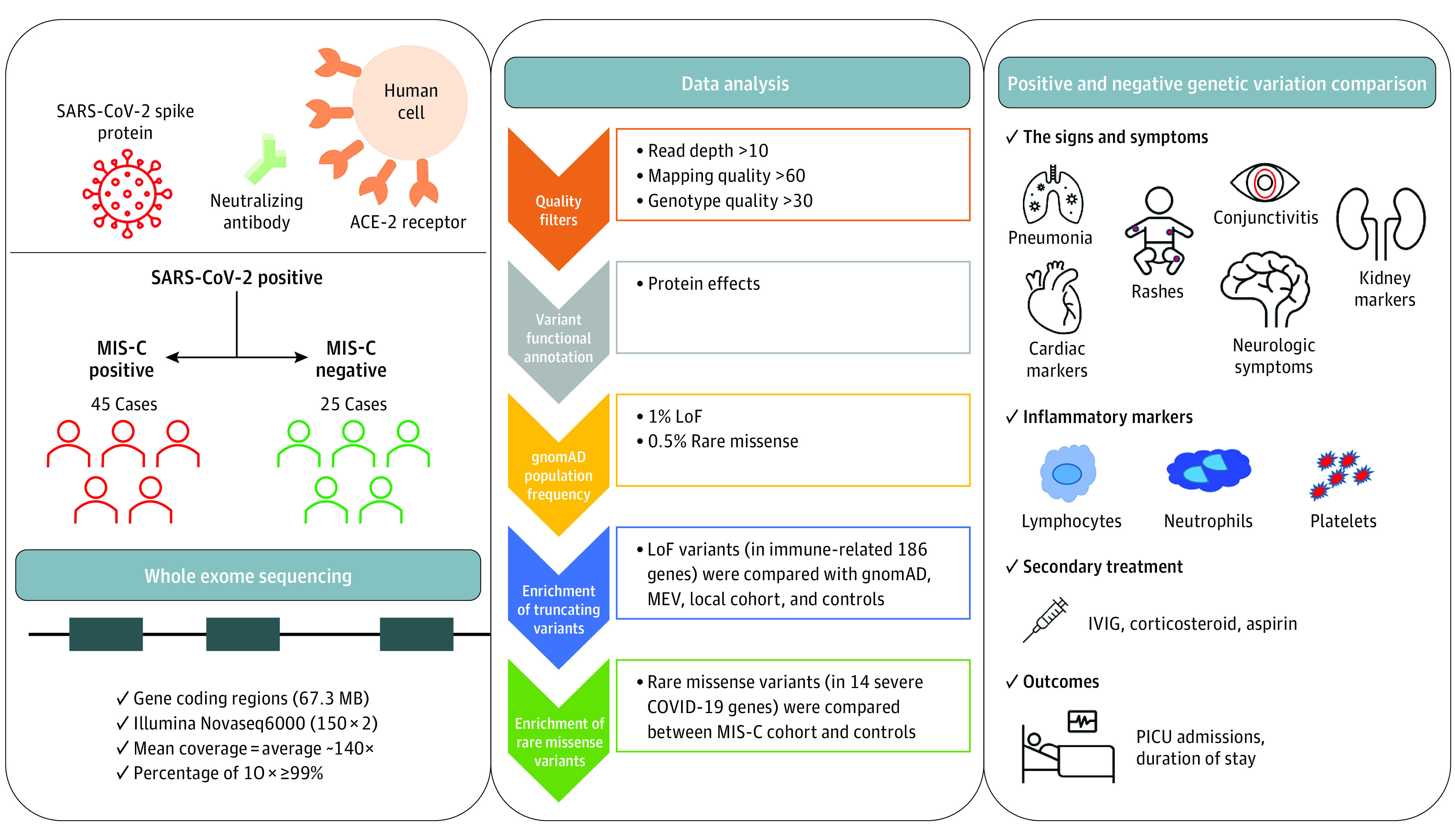
Graphical Representation of Study Design, Participants, Sequencing Protocol, Bioinformatic Analysis, and Genomic and Clinical Characterization of Patients With Multisystem Inflammatory Syndrome in Children (MIS-C) ACE-2 indicates angiotensin-converting enzyme 2; LoF, loss of function; gnomAD, Genome Aggregation Database; MEV, Middle East Variome database; IVIG, intravenous immunoglobulin; and PICU, pediatric intensive care unit.

Whole exome sequencing was performed in the genomic laboratory at Al Jalila Children’s Hospital (Figure 1). DNA was extracted from peripheral blood cells using standard DNA extraction protocols (Qiagen). After fragmentation by ultrasonication (Covaris), genomic DNA was processed to generate sequencing-ready libraries of short fragments (300-400 bp) using the SureSelect kit (Agilent). RNA baits targeting all coding regions were used to enrich for whole exome regions using the SureSelect Clinical Research Exome V2 kit (Agilent). The enriched libraries underwent next-generation sequencing (2 × 150 bp) using the SP flow cell and the NovaSeq platform (Illumina).

### Bioinformatics Analysis and Variant Filtration

Sequencing data were processed using an in-house custom-made bioinformatics pipeline to retain high-quality sequencing reads with greater than 10 × coverage across all coding regions. High-quality variants with read depths greater than 10, genotypic quality greater than 30, and mapping quality greater than 60 were retained for downstream analysis.^20^ We prioritized rare variants in 186 signature genes (eTable 2 in the Supplement) that may be implicated in disease progression of MIS-C, mainly genes that are associated with immune responses, including cellular response to cytokine, cell mediation of immunity, and immune and interferon signaling pathways from reported literature.^21,22,23,24^ We used 3 filters to retain (1) truncating or loss of function (LoF) variants in the 186 inflammatory- or immune-related genes with deleterious effect on Reference Sequence database canonical transcripts and allele frequency less than 1% in the Genome Aggregation Database (gnomAD); (2) homozygous missense or LoF variants across the 186 genes and allele frequency less than 1% in gnomAD; and (3) missense variants in a subset of genes (n = 14) recently associated with severe COVID-19^21,22^ (eTable 2 in the Supplement) and gnomAD allele frequency less than 0.5%. We applied similar filters to whole exome sequencing data from controls (n = 25) and compared these variants between patients with MIS-C and controls.

### Enrichment Analysis

Enrichment of rare, likely deleterious genetic variants in patients with MIS-C was determined by comparing the proportion of individuals with at least 1 functional allele (nonsense, frameshift, missense, and canonical +1 and +2 residues at the 5′ donor splice site and −1 and −2 residues at the 3′ acceptor splice site) in the MIS-C group with that in controls. *P* values were calculated using the Fisher exact test (GraphPad Prism, version 9.2.0; GraphPad Software Inc). Similarly, the number of individuals with more than 1 heterozygous variant (double heterozygotes) were compared between the MIS-C and control groups as another measure of the burden of genetic variation in patients.

Furthermore, to rule out the possibility that the genes affected with truncating or LoF variants in patients with MIS-C were simply tolerant to such variation, we quantified the fractions of such variants in those genes in general populations, namely gnomAD,^25^ Middle East Variome database^26^ (created in-house by assembling sequencing data from Qatar^27^ and the Greater Middle East^28^), and an internal local cohort (exome sequencing data from healthy parents in the United Arab Emirates), and compared these data with those for patients with MIS-C. The aggregate LoF allele fraction was obtained by summing the total LoF allele fraction in each of the above genes (total LoF allele counts divided by maximum allele number in the database). The Fisher exact test was performed using GraphPad Prism software, version 9.2.0.

### Pathway and Protein-Protein Interaction Analysis

String^29^ was used for pathway enrichment and protein-protein interaction (PPI) network analysis. The network nodes correspond to proteins, whereas the edges represent confidence based on available experimental evidence and expert-curated databases. We applied an interaction score of highest confidence (0.9) to avoid any false-positive interactions among proteins.

### Statistical Analysis

We divided patients into the 2 groups based on the presence or absence of genetic variation and performed a Mann-Whitney *U* test to compare clinical and pathologic findings (mucocutaneous, gastrointestinal, and neurologic), inflammatory markers (white blood cells, hemoglobin, C-reactive protein, aspartate aminotransferase, alanine aminotransferase, neutrophils, lymphocytes, platelets, D-dimer, erythrocyte sedimentation rate, albumin, ferritin, fibrinogen, urea, and creatinine), treatment, management, and outcomes (admission to the PICU, intravenous immunoglobulin [IVIG] doses, etc) using GraphPad Prism software, version 9.2.0. We did not impute missing data. Categorical variables were compared using the Fisher exact test. Tests were 2-tailed, and *P* < .05 was considered statistically significant. Given the potential for type I error as a result of multiple comparisons, findings should be interpreted as exploratory.^30^

## Results

### Demographic and Clinical Characteristics of Patients With MIS-C

**Figure 2.**
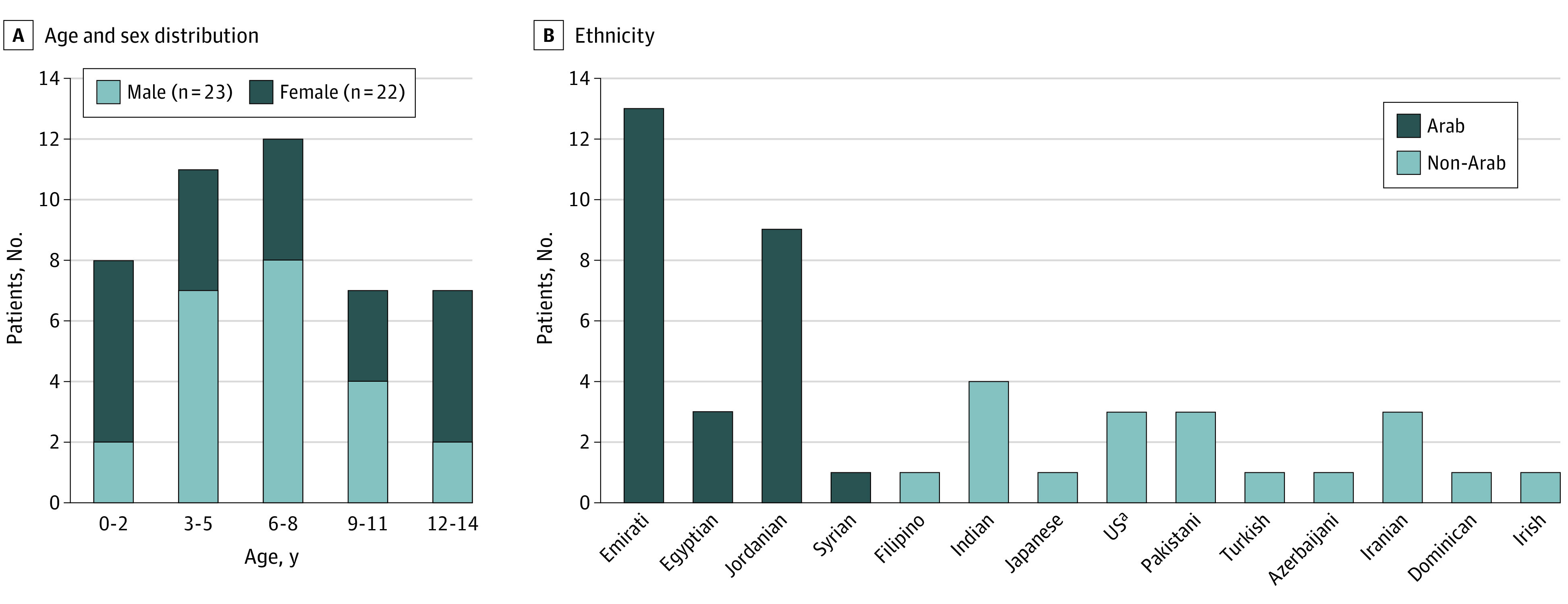
Age, Sex, and Country of Origin of Patients With Multisystem Inflammatory Syndrome in Children ^a^Patients self-identified as being from the US (no other information is available).

A total of 45 patients with MIS-C (23 [51.1%] male; 30 [66.7%] from Middle Eastern countries of origin [United Arab Emirates, Jordan, Iran, Syria, Egypt, and Turkey]; mean [SD] age, 6.7 [3.6] years) (Figure 2) and 25 controls (17 [68.0%] male; 24 [96.0%] from Middle Eastern countries of origin [United Arab Emirates, Jordan, and Palestine] and 1 from the Philippines; mean [SD] age, 7.4 [4.0] years) were prospectively recruited into the cohort (eTable 1 in the Supplement). Thirty-six patients (80.0%) had evidence of SARS-CoV-2 infection by RT-PCR or antibody testing, whereas 9 (20.0%) had exposure to the virus through contact with patients with COVID-19. The mean (SD) duration of fever was 5 (2.4) days (eTables 3 and 4 in the Supplement), and all patients had laboratory evidence of inflammation, including lymphopenia and/or significantly elevated inflammatory markers, such as C-reactive protein, erythrocyte sedimentation rate, D-dimer, ferritin, fibrinogen, and IL-6 (eTable 5 in the Supplement).

Gastrointestinal symptoms and mucocutaneous involvement were the most common clinical findings and were each reported in 36 patients (80.0%; 95% CI, 66.1%-89.1%). Neurologic symptoms were reported in 14 patients (31.1%; 95% CI, 19.5%-45.6%), and respiratory findings were reported in 12 patients (26.6%; 95% CI, 16.0%-41.0%) (eTable 3 in the Supplement). Cardiac involvement was reported in 22 patients (48.9%; 95% CI, 35.0%-63.0%), and 19 patients (42.2%; 95% CI, 30.0%-56.7%) had hypotension and were in shock (eTable 3 in the Supplement). The clinical manifestations in our cohort were consistent with findings in the scientific literature.^31,32^

### Genetic and Enrichment Analysis

**Figure 3.**
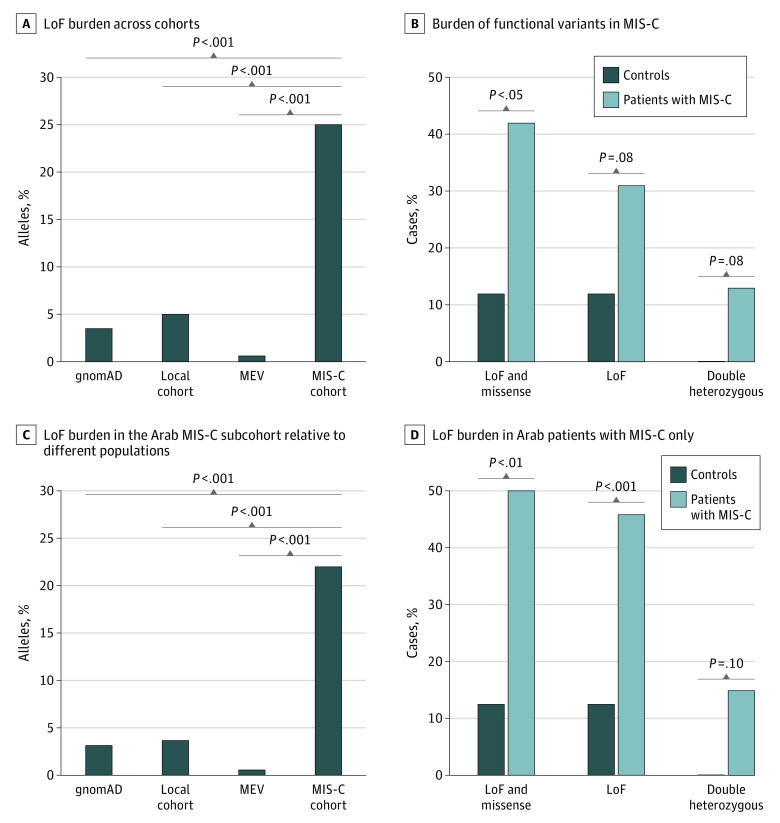
Burden of Immune-Related Loss of Function (LoF) and Missense Variants in Patients With Multisystem Inflammatory Syndrome in Children (MIS-C) gnomAD indicates Genome Aggregation Database; MEV, Middle East Variome database.

We detected 20 truncating heterozygous variants affecting 16 immune-related genes (*CD84* [OMIM 604513], *CD163* [OMIM 605545], *IFI44* [OMIM 610468], *IFI44L* [OMIM 613975], *IFIH1* [OMIM 606951], *IFNA21* [OMIM 147584], *IFNA4* [OMIM 147564], *IFNA6* [OMIM 147566], *IFNB1* [OMIM 147640], *IL22RA2* [OMIM 606648], *IRAK3* [OMIM 604459], *LY9* [OMIM 600684], *NLRP12* [OMIM 609648], *NLRP2* [OMIM 609364], *RAB27A* [OMIM 603868], and *TLR6* [OMIM 605403]) in 14 patients with MIS-C (Table). Those genes were not similarly enriched for truncating variants in the general population as in gnomAD, in a combined set of exome sequencing data from the Greater Middle East variome project (N = 1111)^28^ and a Qatari cohort (N = 1005),^27^ or in our internal cohort of healthy individuals (N = 124) (Figure 3A). Finally, using a similar filtration strategy in the control group, we detected only 3 truncating variants (eTable 6 in the Supplement) across all 186 genes (vs 20 in patients with MIS-C), further confirming the higher burden of such variants in the MIS-C group (Figure 3B). Furthermore, 6 of the 14 patients with MIS-C with truncating variants harbored 2 such variants in 2 different genes (Figure 3B and Table), including 2 patients (COVGEN-7 and COVGEN-36) who were double heterozygous for truncating variants in *IL22RA2* and *IFI44L* and 1 patient (COVGEN-30) with 2 heterozygous variants in *IFNA6* and *IFNA21*. Similar findings were obtained when analysis was focused only on patients with MIS-C who tested positive for SARS-CoV-2 (n = 36) and excluding those with only exposure to COVID-19 (eFigure 2 in the Supplement).

**Table.** Genetic Findings in Patients With Multisystem Inflammatory Syndrome in Children

Case and chromosome coordinates	Gene(s)	Transcript	Complementary DNA	Protein effect	Zygosity	Effect	Disease pathway
COVGEN-27							
chr19:54301595	*NLRP12*	NM_144687.3	c.2828_2829dupTC	p.Arg944Serfs*6	Heterozygous	Frameshift	NOD-like receptor signaling pathway
chr4:38830758	*TLR6*	NM_006068.4	c.337C>T	p.Gln113Ter	Heterozygous	Nonsense	Toll-like receptor signaling pathway
chr19:50163998	*IRF3*	NM_001571.6	c.1070C>T	p.Pro357Leu	Heterozygous	Missense	Toll-like receptor signaling pathway
COVGEN-18							
chr12:66641621	*IRAK3*	NM_007199.3	c.1461delC	p.Asn487Lysfs*10	Heterozygous	Frameshift	IL-1 signaling pathway
chr1:160784531	*LY9*	NM_002348.4	c.1052_1053delAT	p.His351Argfs*22	Heterozygous	Frameshift	IL-2 signaling pathway
chr4:187003729	*TLR3*	NM_003265.3	c.889C>G	p.Leu297Val	Heterozygous	Missense	Toll-like receptor signaling pathway
chr14:103369742	*TRAF3*	NM_003300.4	c.1111G>A	p.Ala371Thr	Heterozygous	Missense	Toll-like receptor signaling pathway
COVGEN-36							
chr6:137479566	*IL22RA2*	NM_052962.3	c.115C>T	p.Arg39Ter	Heterozygous	Nonsense	Cytokine signaling in immune system
	*IFI44L*	NM_006820.4	c.873T>A	p.Tyr291Ter	Heterozygous	Nonsense	Immune response
COVGEN-7							
chr6:137479566	*IL22RA2*	NM_052962.3	c.115C>T	p.Arg39Ter	Heterozygous	Nonsense	Cytokine signaling in immune system
	*IFI44L*	NM_006820.4	c.873T>A	p.Tyr291Ter	Heterozygous	Nonsense	Immune response
COVGEN-6							
chr2:163133953	*IFIH1*	NM_022168.4	c.2016delA	p.Asp673Ilefs*5	Heterozygous	Frameshift	Induction of type 1 interferons and proinflammatory cytokines
COVGEN-23							
chr19:54299165	*NLRP12*	NM_144687.3	c.3046C>T	p.Arg1016*	Heterozygous	Nonsense	NOD-like receptor signaling pathway
COVGEN-29							
chr9:21077466	*IFNB1*	NM_002176.4	c.403G>T	p.Gly135*	Heterozygous	Nonsense	Toll-like receptor signaling pathway
COVGEN-30							
chr9:21350700	*IFNA6*	NM_021002.2	c.187C>T	p.Glu126Ter	Heterozygous	Nonsense	Toll-like receptor signaling pathway
chr9:21166236	*IFNA21*	NM_002175.2	c.376G>T	p.Gln63Ter	Heterozygous	Nonsense	Toll-like receptor signaling pathway
COVGEN-8							
chr2:163136505	*IFIH1*	NM_022168.4	c.1641 + 1G>C	p.?	Heterozygous	NR	Induction of type 1 interferons and proinflammatory cytokines/RIG-I–like receptor signaling pathway
COVGEN-13							
chr1:160535263	*CD84*	NM_003874.4	c.319delT	p.Tyr107Thrfs*5	Heterozygous	Frameshift	Regulation of innate and adaptive immune response
chr12:7635263	*CD163*	NM_203416.4	c.3223C>T	p.Arg1075Ter	Heterozygous	Nonsense	Anti-inflammatory role
COVGEN-16							
chr19:55493698	*NLRP2*	NM_017852.5	c.632dupT	p.Tyr212Valfs*57	Heterozygous	Frameshift	Activation of proinflammatory caspases
chr1:79101171	*TRIM69*	NM_182985.5	c.1404C>A	p.Phe468Leu	Heterozygous	Missense	Antigen processing and presentation
COVGEN-38							
chr15:55516202	*RAB27A*	NM_004580.5	c.352C>T	p.Gln118Ter	Heterozygous	Nonsense	Immune responses
COVGEN-39							
chr1:79121088	*IFI44*	NM_006417.5	c.732C>G	p.Tyr244Ter	Heterozygous	Nonsense	Immune responses
COVGEN-45							
chr9:21187471	*IFNA4*	NM_021068.3	c.60T>A	p.Cys20*	Heterozygous	Nonsense	Toll-like receptor signaling pathway
COVGEN-33							
chr4:187003729	*TLR3*	NM_003265.3	c.889C>G	p.Leu297Val	Heterozygous	Missense	Toll-like receptor signaling pathway
COVGEN-25							
chr15:45059871	*TRIM69*	NM_182985.5	c.1404C>A	p.(Phe468Leu)	Heterozygous	Missense	Antigen processing and presentation
COVGEN-20							
chr21:34632925	*IFNAR2*	NM_001289125.3	c.733G>C	p.Gly245Arg	Heterozygous	Missense	Toll-like receptor signaling pathway
COVGEN-17							
chr21:34632925	*IFNAR2*	NM_001289125.3	c.733G>C	p.Gly245Arg	Heterozygous	Missense	Toll-like receptor signaling pathway
COVGEN-43							
chr21:34635648	*IFNAR2*	NM_001289125.3	c.1391A>C	p.Asn464Thr	Heterozygous	Missense	Toll-like receptor signaling pathway

Abbreviations: IL, interleukin; NR, not reported; RIG-I, retinoic acid–inducible gene I.

We also identified 6 rare missense variants in 9 patients with MIS-C (3 of whom also carried truncating variants from above analysis) in 5 of the 14 genes (*IFNAR2* [OMIM 602376], *IRF3* [OMIM 603734], *TLR3* [OMIM 603029], *TRAF3* [OMIM 608255], and *TRIM69* [OMIIM 616017]) known to cause severe COVID-19 (Table) and none in the control group. Of interest, 1 patient (COVGEN-27) carried 4 rare heterozygous variants (2 truncating and 2 missense) in *IRAK3*, *LY9*, *TLR3*, and *TRAF3*, and 1 patient (COVGEN-27) had 3 variants (2 truncating and 1 missense) in *NLRP12*, *TLR6*, and *IRF3* (Table).

We performed a similar enrichment analysis on the set of patients with MIS-C (n = 26) and controls (n = 24) from Arab countries of origin. There was a higher burden of LoF variants in Arab patients with MIS-C relative to other population databases (Figure 3C). More importantly, there was an enrichment of LoF and missense variants in patients with MIS-C when compared with Arab-matched controls, ruling out a possible genetic background effect (Figure 3D).

**Figure 4.**
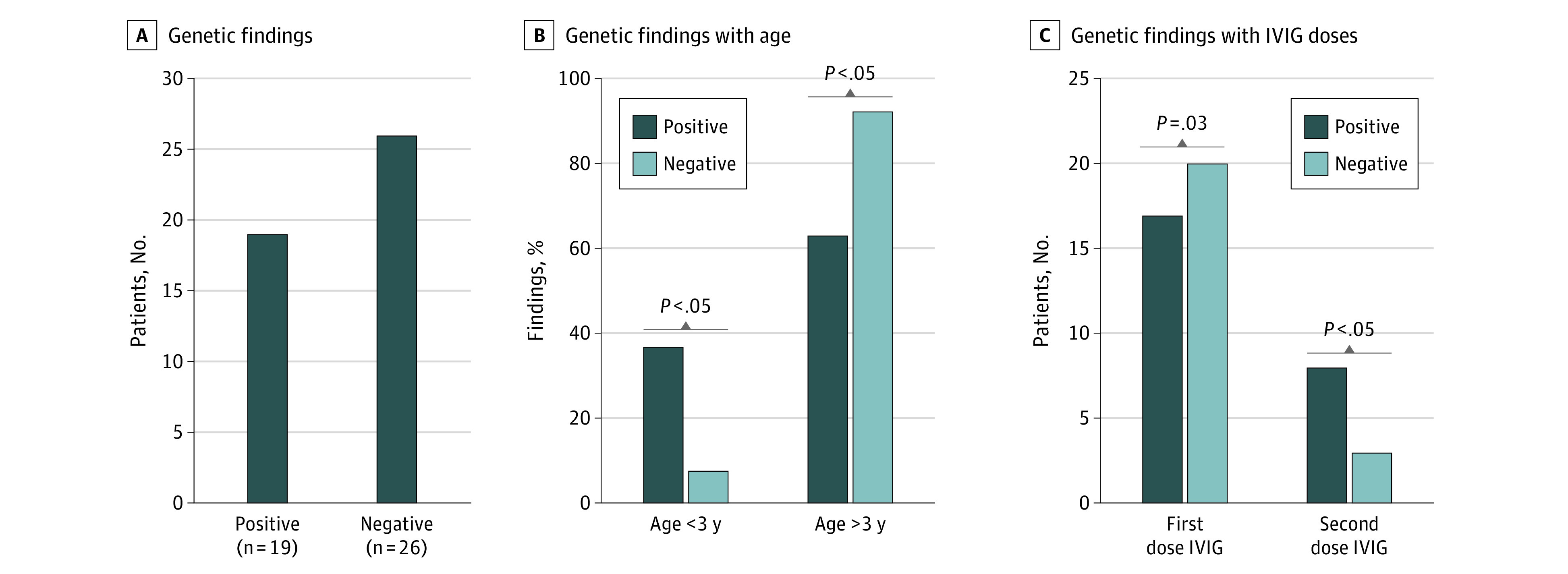
Genetic Findings and Associations With Age and Response to Treatment Among 19 patients with multisystem inflammatory syndrome in children with positive genetic findings, 7 (36.8%) were younger than 3 years, whereas 12 (63.2%) were older than 3 years. Of 26 patients with no genetic variants, 2 (7.7%) were younger than 3 years, whereas 24 (92.3%) were older. Although most patients received 1 dose of intravenous immunoglobulin (IVIG), a significantly higher proportion of patients with genetic findings (8 of 19 [42.1%]) received a second dose compared with those without genetic variants (3 of 26 [11.5%]).

Overall, 19 patients with MIS-C (42.2%; 95% CI, 29.0%-56.7%) had rare truncating or missense variants (Figure 4A), and 7 (36.8%; 95% CI, 19.1%-59.0%) of those patients had more than 1 variant (Figure 3D). Our combined enrichment analysis for both missense and truncating variants showed that patients with MIS-C had a higher burden of such variants relative to the control group.

### Pathway and Protein Network Analysis

Genes with enriched variants belonged to the Toll-like receptor signaling, retinoic acid–inducible gene I–like receptor signaling, interferon-mediated immune response, and NOD-like receptor signaling pathways as the main pathways altered in patients with MIS-C (Table). We obtained a PPI network, based on experimental evidence and expert-curated databases, with 10 nodes representing proteins and 23 edges representing confidence level, with a PPI enrichment *P* < 1.0^−16^. Interactions among *IFNB1, IFNA21, IFNA4, IFNA6, IFNAR2, TRAF3, IRF3,* and *TLR3* indicate the role of *IFN*-mediated immune responses (eFigure 3 in the Supplement).

### Genetic Associations With Clinical and Demographic Characteristics of Patients With MIS-C

We tested the association between the genetic status of patients with MIS-C and their demographic, clinical, and laboratory findings (Figure 1). On the basis of age distribution of patients with MIS-C (eFigure 4 in the Supplement), we observed that patients who were younger than 3 years were more likely to harbor a rare variant in the immune-related genes (7 patients with genetic findings [36.8%; 95% CI, 19.1%-58.9%] vs 2 without genetic findings [7.7%; 95% CI, 2.1%-24.1%] were younger than 3 years at onset; *P* = .02, Fisher exact test) (Figure 4B). No significant clinical association was found between genetic findings and symptoms (eTable 7 in the Supplement). Most laboratory markers were similarly abnormal in all patients with MIS-C; those with a genetic finding had higher fibrinogen levels, but this finding was not statistically significant (eTable 5 in the Supplement). Similarly, no associations were obtained when analysis was focused only on patients with MIS-C who tested positive for SARS-CoV-2 (n = 36) while excluding those with only exposure to COVID-19 (eTables 8 and 9 in the Supplement).

### Management, Hospital Course, and Outcomes

All patients recovered after a mean (SD) length of stay of 6.5 (4.8) days. Nineteen patients (42.2%) were admitted to the PICU. Thirty-seven patients (82.2%) received at least 1 high dose (2 g/kg) of IVIG during 12 hours. Of the 11 patients who received 2 doses of the IVIG, 8 had genetic findings (8 of 19 vs 3 of 26, *P* = .04, 2-tailed Fisher exact test) (Figure 4C; eTable 10 in the Supplement), suggesting a possible resistance to treatment, defined by persistence of fever, worsening in organ dysfunction, and increase in inflammatory markers that required intensification of treatment (second dose of IVIG). Thirty-one patients (68.9%) were given corticosteroids, and 36 (80.0%) were given aspirin. Enoxaparin was received by 9 patients (20.0%). No association was found between genetic status and treatment with corticosteroid, aspirin, enoxaparin, or oxygen intervention. Notably, of the 7 patients who had more than 1 genetic variant, 5 were admitted to the PICU and received intensive care treatment (2 received invasive treatment and 3 received noninvasive ventilatory support), 4 of them had cardiac dysfunction, and all of them had gastrointestinal symptoms. No deaths occurred among the patients recruited in this study.

## Discussion

In this cohort study, we characterize the genomic landscape, phenotypic features, inflammatory and cellular markers, and clinical management and outcomes of the largest prospective cohort to date of patients with MIS-C from the Middle East. To our knowledge, this study is also the largest MIS-C cohort involving whole exome sequencing data. Our cohort included patients who were mostly from the Middle East (66.7%), including 26 from Arab countries and 9 from Asian countries. Patients of such backgrounds have long been underrepresented in genetic studies, emphasizing the importance of our study in characterizing the genetic landscape of MIS-C disease in this cohort. Although clinical presentations and laboratory markers in this cohort were consistent with recently described MIS-C cohorts elsewhere,^7^ our analysis revealed significant enrichment of rare, likely deleterious variants mainly affecting the Toll-like receptor signaling, retinoic acid–inducible gene I–like receptor, NOD-like receptor signaling, and interferon-mediated immune response pathways in patients relative to age- and genetically matched controls and to the general population of diverse origins (including our internal cohort of healthy individuals). Those pathways overlap with the currently characterized immunologic profile in patients with MIS-C discussed in the Introduction.^12,13^

In addition, transcriptomic profiling of patients with severe COVID-19 found upregulation of cytokines and chemokines, including CCL2, CCL22, CXCL9, CCL2/MCP-1, CXCL10/IP-10, CCL3/MIP-1A, and CCL4/MIP1B and *CXCL12*, and certain interferons and interleukins, including *IFIH1*, *IFI44*, *IFIT1*, and *IL10*.^33,34^ Similarly, single-cell transcriptomic profiling of older individuals with SARS-CoV-2 identified type 1 and 2 interferon deficiencies and reduced expression of antiviral defense genes in certain cell types, indicating decreased immune ability in response to SARS-CoV-2 infection in older individuals.^35^ All these studies^33,34,35^ and our current genetic findings indicate possible mechanistic convergence between MIS-C and severe COVID-19 on key immune-related pathways. Nonetheless, although our genetic analysis revealed some overlap with the type 1 IFN pathway, shown to be altered in patients with severe COVID-19,^18^ most of the identified variants, especially truncating ones, in patients with MIS-C affect genes that do not overlap with those in patients with severe COVID-19. These results suggest that MIS-C and severe COVID-19 have distinct genetic determinants while possibly altering similar inflammatory pathways.

Our genetic analysis also revealed that 7 of the 19 patients (36.8) had more than 1 variant, including 2 patients with 4 and 3 variants, respectively, further highlighting the significant burden of genetic variation in this cohort. Despite the relatively high rate of consanguineous marriage in this part of the world, our analysis did not identify any rare homozygous variants that might predispose individuals to MIS-C. It is possible that such variants might lead to severe immune-related disorders irrespective of SARS-CoV-2 infection. Patients with such genetic variants or disorders would have been excluded from our study because almost all our patients did not have significant medical history before viral infection.

### Limitations

This study has some limitations. Our analysis showed that patients with genetic findings tend to have earlier onset of disease (younger than 3 years) and possible resistance to IVIG treatment; however, no significant associations were found between inflammatory markers or clinical symptoms and genetic findings. Such associations cannot be ruled out for several reasons. First, our sample size might not be empowered to detect such associations, and our patient population was primarily of Middle Eastern origin. Second, our study was designed to mainly capture rare, relatively large effect variants in selected genes (N = 186). Therefore, genetic contribution from noncoding variants or deleterious variants outside the 186 studied genes or attributable to polygenic, small-effect variants across the genome cannot be ruled out. Larger sample sizes are still needed to capture such small-effect genetic contributions.

Another limitation of this study is the lack of functional analyses to characterize the mechanism(s) through which the mutated genes contribute to MIS-C disease onset and/or progression. However, most variants are highly concentrated to genes in the IFN and Toll-like receptor pathways, suggesting that disturbance of such pathways might underlie the cytokine storms and dysregulated inflammatory markers in those patients. Protein-protein network analysis further confirms the significant interaction and convergence of most mutated genes in this study.

## Conclusions

The results of this cohort study on patients from the Middle East suggest that MIS-C has a genetic component. This finding paves the way for additional studies designed to include larger numbers of patients from diverse backgrounds along with functional analyses to fully characterize the genetic contribution to this new disease entity.

## Notes

Supplement. **eFigure 1.** Age, Sex, and Origin of the Control Group **eFigure 2**. Burden of Immune-Related LoF and Missense Variants in MIS-C Patients Excluding 9 Patients With Exposures **eFigure 3.** Protein-Protein Interaction (PPI) Network Representation of the Genes With Rare Variants in MIS-C Patients **eFigure 4. **Age Distribution of MIS-C Patients With (Filled Red Circles) or Without (Filled Black Circles) Rare Deleterious Genetic Variants **eTable 1.** MIS-C Associated With COVID-19 **eTable 2.** Immune-Related Genes Used to Filter Rare Coding Variants (Yellow-Highlighted Genes Were Recently Implicated in Severe COVID-19) **eTable 3.** Clinical Characteristics of MIS-C Cohort (N = 45) **eTable 4.** All MIS-C Cases in This Study Along With Evidence of SARS-Cov-2 Infection, Fever, and Inflammatory Markers **eTable 5.** Inflammatory Markers in MIS-C Patients on Admission **eTable 6.** Variants Detected in Control Group **eTable 7.** Clinical and Pathological Findings **eTable 8.** Clinical and Pathological Findings of MIS-C Patients With Positive SARS-CoV-2 Status Only (n=36) **eTable 9.** Inflammatory Markers of MIS-C Patients With Positive SARS-CoV-2 Status Only (n=36) **eTable 10.** Treatment, Management, and Outcomes
