# Supplementary material for: Genetic and Clinical Characteristics of Patients in the Middle East With Multisystem Inflammatory Syndrome in Children
Source: JAMA Netw Open. 2022 May 31;5(5):e2214985. doi: 10.1001/jamanetworkopen.2022.14985 (PMC9157271; doi:10.1001/jamanetworkopen.2022.14985)
Supplement: Supplement. — eFigure 1. Age, Sex, and Origin of the Control Group eFigure 2. Burden of Immune-Related LoF and Missense Variants in MIS-C Patients Excluding 9 Patients With Exposures eFigure 3. Protein-Protein Interaction (PPI) Network Representation of the Genes With Rare Variants in MIS-C Patients eFigure 4. Age Distribution of MIS-C Patients With (Filled Red Circles) or Without (Filled Black Circles) Rare Deleterious Genetic Variants eTable 1. MIS-C Associated With COVID-19 eTable 2. Immune-Related Genes Used to Filter Rare Coding Variants (Yellow-Highlighted Genes Were Recently Implicated in Severe COVID-19) eTable 3. Clinical Characteristics of MIS-C Cohort (N = 45) eTable 4. All MIS-C Cases in This Study Along With Evidence of SARS-Cov-2 Infection, Fever, and Inflammatory Markers eTable 5. Inflammatory Markers in MIS-C Patients on Admission eTable 6. Variants Detected in Control Group eTable 7. Clinical and Pathological Findings eTable 8. Clinical and Pathological Findings of MIS-C Patients With Positive SARS-CoV-2 Status Only (n=36) eTable 9. Inflammatory Markers of MIS-C Patients With Positive SARS-CoV-2 Status Only (n=36) eTable 10. Treatment, Management, and Outcomes [file jamanetwopen-e2214985-s001.pdf]

Supplementary Online Content

Abuhammour W, Yavuz L, Jain R, et al. Genetic and clinical characteristics of patients in the Middle East with multisystem inflammatory syndrome in children. *JAMA Netw Open*. 2022;5(5):e2214985. doi:10.1001/jamanetworkopen.2022.14985

eFigure 1. Age, Sex, and Origin of the Control Group

eFigure 2. Burden of Immune-Related LoF and Missense Variants in MIS-C Patients Excluding 9 Patients With Exposures

eFigure 3. Protein-Protein Interaction (PPI) Network Representation of the Genes With Rare Variants in MIS-C Patients

eFigure 4. Age Distribution of MIS-C Patients With (Filled Red Circles) or Without (Filled Black Circles) Rare Deleterious Genetic Variants

eTable 1. MIS-C Associated With COVID-19

eTable 2. Immune-Related Genes Used to Filter Rare Coding Variants (Yellow-Highlighted Genes Were Recently Implicated in Severe COVID-19)

eTable 3. Clinical Characteristics of MIS-C Cohort (N = 45)

eTable 4. All MIS-C Cases in This Study Along With Evidence of SARS-Cov-2 Infection, Fever, and Inflammatory Markers

eTable 5. Inflammatory Markers in MIS-C Patients on Admission

eTable 6. Variants Detected in Control Group

eTable 7. Clinical and Pathological Findings

eTable 8. Clinical and Pathological Findings of MIS-C Patients With Positive SARS-CoV-2 Status Only (n=36)

eTable 9. Inflammatory Markers of MIS-C Patients With Positive SARS-CoV-2 Status Only (n=36)

eTable 10. Treatment, Management, and Outcomes

This supplementary material has been provided by the authors to give readers additional information about their work.

© 2022 Abuhammour W et al. *JAMA Network Open.*

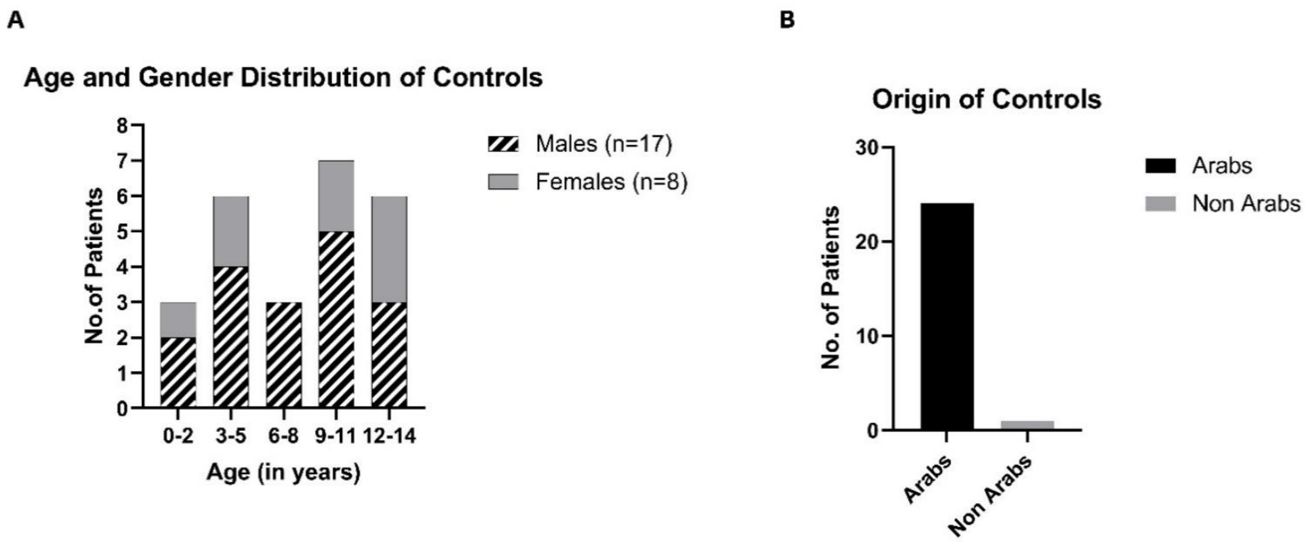

eFigure 1. Age, Sex, and Origin of the Control Group

A, Age and gender distribution of Controls, x-axis represents age (in years), and y-axis represents number of patients. Grey filled areas represent females while dashed areas represent males. B, Origin of individuals in the Control group, x- axis shows origin while y-axis shows number of patients. Black filled bars represent Arabs while grey filled bars represent non-Arabs.

© 2022 Abuhammour W et al. *JAMA Network Open.*

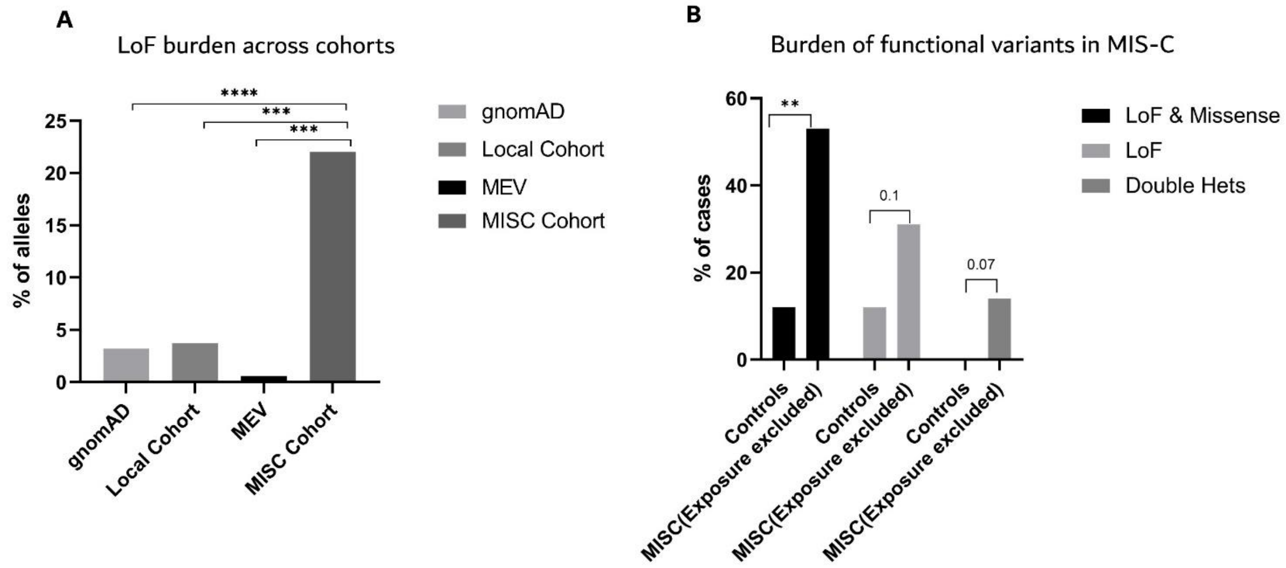

Figure 2. Burden of Immune-Related LoF and Missense Variants in MIS-C Patients Excluding 9 Patients With Exposures

A, Frequency of truncating variants in the 16 genes identified in MIS-C cohort relative to different populations, y axis representing % of alleles. **B**, Proportion of individuals with LoF variants, LoF and Missense variants, and number of double heterozygotes in the control group and MIS-C patients, y-axis represents % of cases. **$P < .01$; ***$P < .001$; ****$P < .0001$ by the Mann-Whitney test.

© 2022 Abuhammour W et al. *JAMA Network Open.*

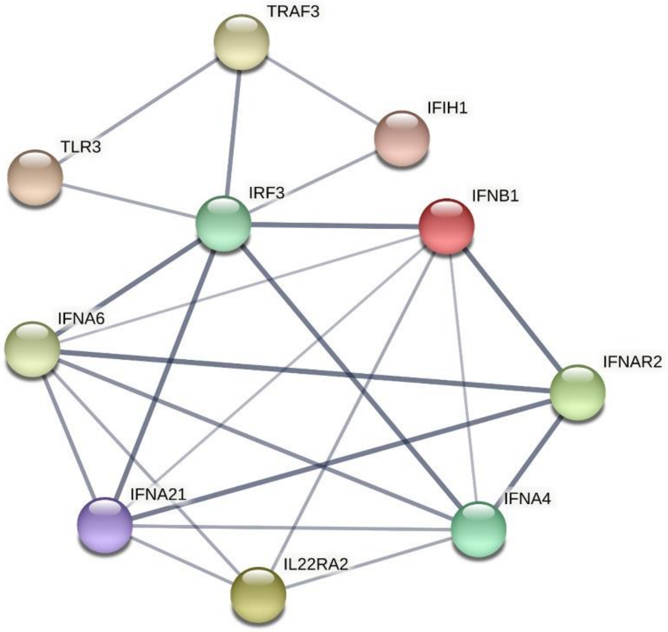

eFigure 3. Protein-Protein Interaction (PPI) Network Representation of the Genes With Rare Variants in MIS-C Patients

Nodes represent proteins, and edges represent confidence of interaction based on functional and curated evidence (See Methods).

© 2022 Abuhammour W et al. *JAMA Network Open.*

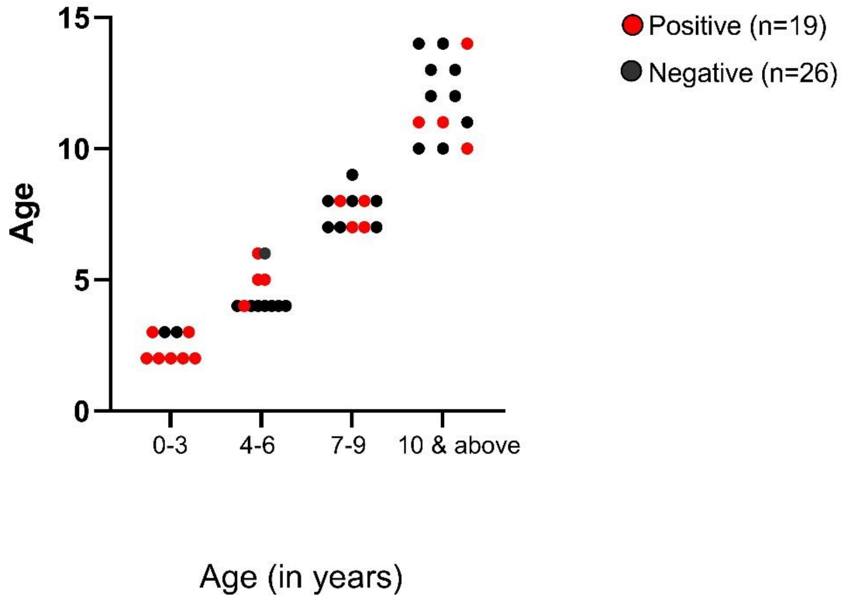

eFigure 4. Age Distribution of MIS-C Patients With (Filled Red Circles) or Without (Filled Black Circles) Rare Deleterious Genetic Variants

As can be seen, majority of those below 3 years of age had positive genetic findings.

© 2022 Abuhammour W et al. *JAMA Network Open.*

**eTable 1.** MIS-C Associated With COVID-19			
Organisation or publication	WHO	US Centre for Disease Control and Prevention	Royal College of Paediatrics and Child Health
Age group	0–19 years	<21 years	Child (age not specified)
Inflammation	Fever and elevated inflammatory markers for 3 days or more	Fever and elevated inflammatory markers	Fever and elevated inflammatory markers
Main features	1. Rash or bilateral non-purulent conjunctivitis or muco-cutaneous inflammation signs (oral, hands or feet).<br>2. Hypotension or shock.<br>3. Features of myocardial dysfunction, pericarditis, valvulitis, or coronary abnormalities (including ECHO findings or elevated Troponin/NT-proBNP),<br>4. Evidence of coagulopathy (by PT, PTT, elevated d-Dimers).<br>5. Acute gastrointestinal problems (diarrhoea, vomiting, or abdominal pain).	Clinically severe illness requiring hospitalisation; and multisystem (two or more) organ involvement (cardiac, renal, respiratory, haematological, gastrointestinal, dermatological, or neurological)	Single or multiple organ dysfunction (shock or respiratory, renal, gastrointestinal, or neurological disorder; additional features
SARS-CoV-19	Evidence of COVID-19 (RT-PCR, antigen test or serology positive), or likely contact with patients with COVID-19.	Other plausible alternative diagnoses Positive RT-PCR, serology, or antigen test; or COVID-19 exposure within the past 4 weeks before symptom onset	RT-PCR positive or negative
Exclusion	Other microbial cause of inflammation	Other plausible alternative diagnoses	Any other microbial cause

© 2022 Abuhammour W et al. *JAMA Network Open.*

eTable 2. Immune-Related Genes Used to Filter Rare Coding Variants (Yellow-Highlighted Genes Were Recently Implicated in Severe COVID-19)

ACP5
ACTB
ADA2
ADAM17
ADAR
AOX1
AP1S3
AP3B1
CAPS
CARD14
CARD8
CASP1
CASP3
CD163
CD27
CD40
CD84
CECR1
CGAS
COPA
CRP
CTLA4
CXCL9
ELANE
FOXP3
GLI3
GSDMD
GZMA
IFI16

© 2022 Abuhammour W et al. *JAMA Network Open.*

IFI27
IFI27L1
IFI27L2
IFI35
IFI44
IFI44L
IFI6
IFIH1
IFIT1
IFIT1B
IFIT2
IFIT3
IFIT5
IFITM1
IFITM10
IFITM2
IFITM3
IFITM5
IFN1
IFNA1
IFNA10
IFNA11P
IFNA12P
IFNA13
IFNA14
IFNA16
IFNA17
IFNA2
IFNA21
IFNA22P

IFNA4
IFNA5
IFNA6
IFNA7
IFNA8
IFNAR1
IFNAR2
IFNB1
IFNE
IFNG
IFNGR1
IFNGR2
IFNK
IFNL1
IFNL2
IFNL3
IFNL4
IFNLR1
IFNR
IFRD1
IFRD2
IKBKG
IL10
IL17A
IL18
IL1A
IL1B
IL1RL2
IL1RN
IL22RA2

© 2022 Abuhammour W et al. *JAMA Network Open.*

IL36RN
IL3RA
IL6
IRAK2
IRAK3
IRAK4
IRF1
IRF2
IRF2BP1
IRF2BP2
IRF2BPL
IRF3
IRF4
IRF5
IRF6
IRF7
IRF8
IRF9
ISG15
ISG20
ITGAX
KLRD1
LPIN2
LY9
LYST
MEFV
MVK
MYBL1
MYD88
NFKB1

© 2022 Abuhammour W et al. *JAMA Network Open.*

NLRC3
NLRC4
NLRP1
NLRP12
NLRP2
NLRP3
NOD2
OTULIN
PAPPA
PLCG2
PMAIP1
POLA1
PRF1
PSMB8
PSTPIP1
PSTPIP2
PTPN18
PTPN6
PYCARD
RAB27A
RAB6A
RIPK1
RNASEH2A
RNASEH2B
RNASEH2C
RNF31
S100A1
S100B
S1PR5
SAMHD1

© 2022 Abuhammour W et al. *JAMA Network Open.*

SH2D1B
SH3BP2
SLC29A3
SLPI
SOCS1
STAT1
STAT2
STIM1
STING1
STX11
STXBP2
TBK1
TBX21
TGFBR3
TICAM
TICAM1
TLR3
TLR4
TLR6
TLR7
TMEM173
TNF
TNFAIP3
TNFRSF1A
TNFRSF9
TRAF3
TREX1
TRNT1
UNC13D
UNC93B1

© 2022 Abuhammour W et al. *JAMA Network Open.*

USP18
WDR1
XIAP
TRIF
NEMO
TRFA3
TICAM3

© 2022 Abuhammour W et al. *JAMA Network Open.*

eTable 3. Clinical Characteristics of MIS-C Cohort (N = 45)

Average age in years (range)	7 (2-14)
Fever (%)	100%
Evidence of SARS-CoV-2 (PCR, antibodies, exposure) (%)	100%
Raised inflammatory markers (CRP, PCT, ESR) (%)	100%
Rash or bilateral non-purulent conjunctivitis or muco-cutaneous inflammatory signs (%, N)	80%, 36
Shock (%, N)	42%, 19
Myocardial dysfunction, pericarditis, valvulitis, or coronary abnormalities (including ECHO findings or elevated Troponin/NT-proBNP) (%, N)	49%, 22
Acute gastrointestinal problems (%, N)	80%, 36
Neurological symptoms (%, N)	31%, 14
Respiratory symptoms (%, N)	26.6%, 12
Kidney Markers (%, N)	42%, 19

Abbreviations: Acute gastrointestinal problems (vomiting, abdominal pain, diarrhea). Neurological symptoms (headache, blurred vision, Papilledema, meningitis, MRI abnormalities). Respiratory symptoms (pneumonia or pleural effusion). Kidney Markers (US abdomen)

© 2022 Abuhammour W et al. *JAMA Network Open.*

eTable 4. All MIS-C Cases in This Study Along With Evidence of SARS-Cov-2 Infection, Fever, and Inflammatory Markers

Case ID	Evidence of SARS-CoV-2			Fever (Duration in days)	Raised inflammatory markers (CRP, PCT, ESR)
	Positive PCR	Positive antibodies	Exposure		
COVGEN-1	No	Yes	Yes	3	Yes
COVGEN-2	Yes	Yes	Yes	5	Yes
COVGEN-3	Yes	Yes	Yes	4	Yes
COVGEN-4	No	Yes	Yes	6	Yes
COVGEN-5	No	Yes	Yes	6	Yes
COVGEN-6	No	Yes	Yes	5	Yes
COVGEN-7	Yes	Yes	Yes	5	Yes
COVGEN-8	No	Yes	Yes	3	Yes
COVGEN-9	No	Yes	Yes	3	Yes
COVGEN-10	No	Yes	Yes	3	Yes
COVGEN-11	N/A	Yes	Yes	4	Yes
COVGEN-12	No	Yes	Yes	6	Yes
COVGEN-13	Yes	Yes	Yes	3	Yes
COVGEN-14	No	Yes	Yes	7	Yes
COVGEN-15	No	Yes	Yes	5	Yes
COVGEN-16	Yes	Yes	Yes	3	Yes
COVGEN-17	No	Yes	Yes	7	Yes
COVGEN-18	Yes	Yes	Yes	3	Yes
COVGEN-19	No	No	Yes	9	Yes
COVGEN-20	No	Yes	Yes	6	Yes
COVGEN-21	No	No	Yes	2	Yes
COVGEN-22	No	No	Yes	4	Yes
COVGEN-23	No	No	Yes	3	Yes
COVGEN-24	Yes	N/A	Yes	5	Yes
COVGEN-25	Yes	N/A	Yes	2	Yes
COVGEN-26	No	No	Yes	10	Yes
COVGEN-27	Yes	Yes	No	4	Yes
COVGEN-28	No	Yes	No	10	Yes
COVGEN-29	No	No	Yes	4	Yes
COVGEN-30	No	No	Yes	7	Yes
COVGEN-31	Yes	Yes	Yes	3	Yes
COVGEN-32	N/A	Yes	N/A	5	Yes
COVGEN-33	No	Yes	Yes	7	Yes
COVGEN-34	N/A	Yes	N/A	5	Yes
COVGEN-35	Yes	Yes	Yes	2	Yes
COVGEN-36	N/A	Yes	Yes	4	Yes
COVGEN-37	Yes	N/A	N/A	0	Yes
COVGEN-38	No	Yes	No	11	Yes
COVGEN-39	N/A	Yes	Yes	2	Yes

COVGEN-40	Yes	N/A	No	14	Yes
COVGEN-41	No	No	Yes	4	Yes
COVGEN-42	No	Yes	Yes	3	Yes
COVGEN-43	No	N/A	Yes	15	Yes
COVGEN-44	No	Yes	Yes	5	Yes
COVGEN-45	No	Yes	Yes	2	Yes

© 2022 Abuhammour W et al. *JAMA Network Open.*

eTable 5. Inflammatory Markers in MIS-C Patients on Admission

	Positive genetic variation			Negative genetic variation				Total	
	No. of patients	Mean	Standard Deviation	No. of patients	Mean	Standard Deviation	*P*-value	Total No. of patients	Mean
WBC count, × 10^3 cells/µL (normal range, 5-15 × 10^3 cells/µL)	19	12.48	7.46	26	10.24	5.5	0.4	45	11.18
Hemoglobulin level, mg/dL (normal range, 11-14 mg/dL)	19	10.87	1.58	26	11.09	1.4	0.7	45	11
Absolute neutrophils count, × 10^3cells/µL (normal range, 1.0-8 × 10^3 cells/µL	19	9.2	6.18	24	7.4	5.07	0.3	43	8.23
Absolute lymphocyte count, × 10^3cells/µL (normal range, 4.00-9.00 × 10^3cells/µL)	19	1.7	1.04	24	2.8	2.4	0.5	43	2.30
CRP, mg/dL (normal range, 0-2.8 mg/dL)	19	117.8	107	23	124.7	110.6	0.9	42	121.59
ESR, mm/hr (normal range, <10 mm/hr)	15	58.27	35	22	46.64	20	0.3	37	51.35
Procalcitonin ng/mL (normal range, 0-0.50 ng/mL)	15	10.93	14.6	22	7.7	18.1	0.1	37	8.9
D- dimer, ug/mL (normal range, 0-0.50 ug/mL)	18	4	3	21	2.6	1.99	0.2	39	3.27
Ferritin, ng/mL (normal range, 6-67 ng/mL)	18	571.9	468	22	488	394.6	0.4	40	526

© 2022 Abuhammour W et al. *JAMA Network Open.*

Fibrinogen, mg/dl (normal range, 162-401 mg/dL)	15	603	196	18	476	131	**0.02**	33	533.96
ALT, U/L (normal range, 0-39 U/L)	17	53.9	32.66	22	73.9	169.1	0.1	39	64.89
AST, U/L (normal range, 0-51 U/L)	14	60.71	52.42	21	51.90	94.72	**0.06**	35	55.42
Urea, mg/dL (normal range, 11-36 mg/dL)	17	32.47	23.5	24	28.22	19.65	0.5	41	29.05
Crea, mg/dL (normal range, 0-0.4mg/dL)	17	0.56	0.37	25	0.48	0.18	0.3	42	0.5
ALB, g/dL (normal range, 3.8-5.4 g/dL)	15	3.2	0.49	21	3.3	0.6	0.7	36	3.30
	**No. of patients**	**Median**	**IQR**	**No. of patients**	**Median**	**IQR**		**No. of patients**	**Median**
Platelet count, $\times 10^3$cells/μL (normal range, 200s-490 × 10^3cells/μL)	19	232	241	26	160	166.75	0.4	45	177
Pro-B NP, pg/mL (normal range, < 320 pg/mL)	10	4468	12254	18	1125	3117	0.2	28	1531.5
IL6, pg/mL (normal range, N< 7 pg/mL)	13	81	331	17	46	116	0.1	30	58.65

Abbreviation: No, number of patients. WBC, White Blood Cells. CRP, C-Reactive Protein. ESR, Erythrocyte sedimentation rate. ALT, Alanine Aminotransferase. AST, Aspartate Aminotransferase. ALB, Albumin. IL6, Interleukin 6. IQR, Interquartile range.

© 2022 Abuhammour W et al. *JAMA Network Open.*

eTable 6. Variants Detected in Control Group

Case ID	Chromosome coordinates	Gene(s)	Transcript	cDNA	Protein Effect	Zygosity	Effect
Control-1	chr19:39760612	*IFNL2*	NM_172138.2	c.562C>T	p.Arg188Ter	Het	Stop gained
Control-2	chr19:39760612	*IFNL2*	NM_172138.2	c.562C>T	p.Arg188Ter	Het	Stop gained
Control-3	chr2:163136505	*IFIH1*	NM_022168.4	c.1641+1G>C	p.?	Het	Splice donor variant

© 2022 Abuhammour W et al. *JAMA Network Open.*

eTable 7. Clinical and Pathological Findings

Characteristic	Positive genetic variation (n=19)	Negative genetic variation (n=26)	Total patients (n=45)
Mucocutaneous, N (% within group), 95%CI	14 (73%), 20% to 50%	22(84.6%), 40% to 70%	36 (80%)
Gastrointestinal symptoms, N (% within group), 95%CI	16 (84%), 20% to 60%	20 (77%), 30% to 70%	36 (80%)
Neurological symptoms, N (% within group), 95%CI	8 (42%), 30% to 80%	6 (23%), 10% to 60%	14 (31%)

Abbreviation: mucocutaneous findings (skin rash, inflammation of oral mucosa, conjunctivitis, and extremity findings, including edema of hands and feet. Gastrointestinal symptoms (vomiting, abdominal pain, diarrhea). Neurological symptoms (headache, blurred vision, Papilledema, meningitis, MRI abnormalities).

© 2022 Abuhammour W et al. *JAMA Network Open.*

eTable 8. Clinical and Pathological Findings of MIS-C Patients With Positive SARS-CoV-2 Status Only (n=36)

**Characteristic**	**Positive genetic variation (n=15)**	**Negative genetic variation (n=21)**	**Total patients (n=36)**
Mucocutaneous,<br>N (% within group), 95%CI	10/15 (66%), 40% to 90%	18/21 (86%), 70% to 100%	28 (77.7%)
Gastrointestinal symptoms,<br>N (% within group), 95%CI	13/15 (87%), 70% to 100%	17/21 (81%), 60% to 90%	30 (83%)
Neurological symptoms,<br>N (% within group), 95%CI	7/15 (47%), 20% to 100%	5/21 (24%), 5% to 40%	12 (33%)

Abbreviation: mucocutaneous findings (skin rash, inflammation of oral mucosa, conjunctivitis, and extremity findings, including edema of hands and feet. Gastrointestinal symptoms (vomiting, abdominal pain, diarrhea). Neurological symptoms (headache, blurred vision, Papilledema, meningitis, MRI abnormalities).

© 2022 Abuhammour W et al. *JAMA Network Open.*

eTable 9. Inflammatory Markers for of MIS-C Patients With Positive SARS-CoV-2 Status Only (n=36)

	**Positive genetic variation**			**Negative genetic variation**				
	**No. of patients**	**Mean**	**Standard Deviation**	**No. of patients**	**Mean**	**Standard Deviation**	**Statistical significance**	**No. of Patients**
WBC count, × 10^3 cells/µL (normal range, 5-15 × 10^3 cells/µL)	15	9.2	3.9	21	8.6	4.4	0.5	36
Hemoglobulin level, mg/dL (normal range, 11-14 mg/dL)	15	10.8	1.7	21	11.29	1.4	0.3	36
Absolute neutrophils count, × 10^3cells/µL (normal range, 1.0-8 × 10^3 cells/µL	15	6.8	3.7	19	6.6	4.3	0.6	34
Absolute lymphocyte count, × 10^3cells/µL (normal range, 4.00-9.00 × 10^3cells/µL)	15	1.4	0.5	19	1.8	1.4	0.8	34
CRP, mg/dL (normal range, 0-2.8 mg/dL)	15	100.6	10.4.2	18	142.8	115.8	0.4	33
ESR, mm/hr (normal range, <10 mm/hr)	12	50.92	35.87	17	44.12	18.57	0.9	29
Procalcitonin ng/mL (normal range, 0-0.50 ng/mL)	11	13.27	16.48	17	9.08	20.38	0.3	28
D- dimer, ug/mL (normal range, 0-0.50 ug/mL)	14	4.2	3.005	16	2.5	1.8	0.18	30
Ferritin, ng/mL	14	659.8	498.6	17	552.2	428.2	0.2	31

© 2022 Abuhammour W et al. *JAMA Network Open.*

(normal range, 6-67 ng/mL)								
Fibrinogen, mg/dl (normal range, 162-401 mg/dL)	11	581.9	213.4	13	475.6	146.6	0.2	27
ALT, U/L (normal range, 0-39 U/L)	13	59.23	31.08	17	88.76	190.8	0.08	30
AST, U/L (normal range, 0-51 U/L)	10	54.30	28.50	16	60.44	107.7	0.08	26
Urea, mg/dL (normal range, 11-36 mg/dL)	13	35.77	25.81	17	27.81	22.19	0.4	30
Crea, mg/dL (normal range, 0-0.4mg/dL)	13	0.6	0.4	18	0.5	0.1	0.9	33
ALB, g/dL (normal range, 3.8-5.4 g/dL)	11	3.08	0.43	16	3.3	0.56	0.3	27
	**No. of patients**	**Median**	**IQR**	**No. of patients**	**Median**	**IQR**	**Total No. of patients**	**Median**
Platelet count, $\times 10^3$cells/μL (normal range, 200s-490 × 10^3cells/μL)	15	181	127	21	165	195	36	179
Pro-B NP, pg/mL (normal range, < 320 pg/mL)	10	1039	2680	12	886.5	3938	22	1039
IL6, pg/mL (normal range, N< 7 pg/mL)	11	73	214	14	55.75	25	106.6	214.1

Abbreviation: No, number of patients. WBC, White Blood Cells. CRP, C-Reactive Protein. ESR, Erythrocyte sedimentation rate. ALT, Alanine Aminotransferase. AST, Aspartate Aminotransferase. ALB, Albumin. IL6, Interleukin 6. IQR, Interquartile range.

© 2022 Abuhammour W et al. *JAMA Network Open.*

eTable 10. Treatment, Management, and Outcomes

	**Positive genetic Variation (N = 19)**	**Negative genetic Variation (N = 26)**	**All (% of total 45 patients)**
Admission to PICU,<br>N (% within group), 95%CI	9 (47.3%), 20% to 69%	10 (38.4%), 30% to 70%	19 (42.2%)
Oxygen Invasive,<br>N (% within group), 95%CI	4 (21%), 10 to 70%	5 (26%), 20 to 80%	9 (20%)
Oxygen Non-Invasive,<br>N (% within group, 95%CI	5 (26%), 20% to 80%	4 (15%), 10% to 70%	9 (20%)
First dose IVIG,<br>N (% within group), 95%CI	17 (89%), 20% to 60%	20 (77%), 30% to 70%	37 (82%)
**Second dose IVIG,**<br>**N (% within group)**, 95%CI	**8 (42%), 40% to 90%**	**3 (5.7%), 0% to 50 %**	**11 (24%)**
Aspirin, N (% within group), 95%CI	15 (79%), 20% to 50%	21 (80%), 40% to 70%	36 (80%)
Corticosteroid,<br>N (% within group), 95%CI	15 (79%), 30% to 60%	16 (61%), 30% to 60%	31 (69%)
Enoxaparin,<br>N (% within group), 95%CI	4 (21%), 10% to 70%	5 (19%), 20% to 80%	9 (20%)
Deceased,<br>N (% within group), 95%CI	0%	0%	
	**Mean S. D**	**Mean S. D**	
Duration of the Stay,<br>95%CI	7 4.39	6.19 5.26	44 (98%)

Abbreviations: PICU, Pediatric Intensive Care Unit. IVIG, Intravenous immune globulin.

© 2022 Abuhammour W et al. *JAMA Network Open.*
